# Exosomes for angiogenesis induction in ischemic disorders

**DOI:** 10.1111/jcmm.17689

**Published:** 2023-02-14

**Authors:** Kasra Moeinabadi‐Bidgoli, Malihe Rezaee, Nikoo Hossein‐Khannazer, Amirhesam Babajani, Hamid Asadzadeh Aghdaei, Mandana Kazem Arki, Siamak Afaghi, Hassan Niknejad, Massoud Vosough

**Affiliations:** ^1^ Basic and Molecular Epidemiology of Gastroenterology Disorders Research Center Shahid Beheshti University of Medical Sciences Tehran Iran; ^2^ School of Medicine Shahid Beheshti University of Medical Sciences Tehran Iran; ^3^ Gastroenterology and Liver Diseases Research Center, Research Institute for Gastroenterology and Liver Diseases Shahid Beheshti University of Medical Sciences Tehran Iran; ^4^ Oncopathology Research Center Iran University of Medical Sciences Tehran Iran; ^5^ Prevention of Metabolic Disorders Research Center, Research Institute for Endocrine Sciences Shahid Beheshti University of Medical Sciences Tehran Iran; ^6^ Department of Regenerative Medicine, Cell Science Research Center Royan Institute for Stem Cell Biology and Technology, ACECR Tehran Iran; ^7^ Experimental Cancer Medicine, Institution for Laboratory Medicine Karolinska Institute Stockholm Sweden

**Keywords:** angiogenesis, exosomes, hypoxia, microRNA, physiology, regenerative medicine, stem cells

## Abstract

Ischaemic disorders are leading causes of morbidity and mortality worldwide. While the current therapeutic approaches have improved life expectancy and quality of life, they are unable to “cure” ischemic diseases and instate regeneration of damaged tissues. Exosomes are a class of extracellular vesicles with an average size of 100–150 nm, secreted by many cell types and considered a potent factor of cells for paracrine effects. Since exosomes contain multiple bioactive components such as growth factors, molecular intermediates of different intracellular pathways, microRNAs and nucleic acids, they are considered as cell‐free therapeutics. Besides, exosomes do not rise cell therapy concerns such as teratoma formation, alloreactivity and thrombotic events. In addition, exosomes are stored and utilized more convenient. Interestingly, exosomes could be an ideal complementary therapeutic tool for ischemic disorders. In this review, we discussed therapeutic functions of exosomes in ischemic disorders including angiogenesis induction through various mechanisms with specific attention to vascular endothelial growth factor pathway. Furthermore, different delivery routes of exosomes and different modification strategies including cell preconditioning, gene modification and bioconjugation, were highlighted. Finally, pre‐clinical and clinical investigations in which exosomes were used were discussed.

## 1 BACKGROUND

Ischemic disorders are the result of insufficiency in blood supply, leading to limited oxygen and nutrient transfer. Ischemia could involve most of the organs/tissues including the heart, brain, peripheral vessels, limbs, skin, retina, intestine and kidney.^1^, ^2^ Ischemic diseases are the leading cause of disability and mortality which impose an enormous burden on human healthcare systems worldwide.^3^ Although current therapies for reperfusion including thrombolytic drugs, using vasodilator,^4^, ^5^ surgical bypass, and endovascular intervention,^6^ have shown significant benefits in the treatment of the ischemic damage, however, these therapies often are not optimal for remodelling vascular beds, thereby ischemic diseases remain the leading cause of long‐term disability. In addition, reperfusion has been found to be able to induce subsequent injury in ischemic tissue, a phenomenon termed ischemia–reperfusion (I/R) injury, which is a critical therapeutic challenge.^7^ Besides, ischemia‐induced and I/R‐mediated injuries could lead to fibrosis and dysfunction of the damaged tissues in a long‐time period.^8^, ^9^ Altogether, researchers have leaned towards finding the therapeutic strategies which could stimulate and enhance the regeneration of the ischemic tissues.

Ischemic disorders are contributed by vascular dysfunction, endothelial cell function impairment, vascular integrity deterioration and enhanced expression of adhesion molecules and inflammation mediators.^10^ It is estimated that more than 500 million people worldwide will benefit from the treatment of the ischemic disorders.^11^

Cell‐based therapies are a revolutionary approach that have raised hopes for the treatment of ischemic disorders through various mechanisms such as angiogenesis induction, apoptosis inhibition and blocking inflammatory process. Stem cells are the most frequently used cells in cell‐based therapies, with properties including differentiation capacity, self‐renewal ability and secretion of beneficial paracrine factors. Stem cells have been shown to induce angiogenesis and provide blood supply in the ischemia‐damaged organs.^12^, ^13^ Although cell‐based therapies have shown promising results for treating various ischemic diseases, they are associated with multiple hindrances including low cell survival in the host's tissue and high expenses which emphasize the need for improving cell‐based therapy strategies.^14^ It has been shown that most transplanted cells could not survive more than 4 days post‐transplantation.^15^ It has been reported that <1% of systemic administered mesenchymal stem cells (MSCs) differentiate into the functional cells in the target tissue and a vast majority of them are trapped in the lung and liver. It is believed that transplanted cells exert their therapeutic effects and participate in angiogenesis via their paracrine activity.^16^

Extracellular vesicles (EVs), main agents in cellular paracrine activity, are micro‐ and nano‐sized vesicles which contain bioactive agents and are released by roughly all cells through fusion of multivesicular bodies with the plasma membrane and subsequent release to the intracellular space. Exosomes are a subgroup of EVs with an average size of 30–150 nm.^17^, ^18^, ^19^ Exosomes are considered long‐range intercellular communication tools that transfer various molecules including proteins, DNA, long non‐coding RNAs (lncRNAs), message RNAs (mRNAs) and microRNAs (miRs) to the recipient cells. Exosomal content represents the conditional and functional situation of the parent cell.^20^, ^21^ They easily pass vascular barriers (such as blood brain barrier) due to the nanoscale size and do not have any risk of tumorigenicity formation. Exosomes possess low immunogenicity as they lack the expression of major histocompatibility complex (MHC). Exosomes have a long‐term storage capacity and could be stored at −20°C for months while their biological activity is preserved.^22^, ^23^

Exosomes are used as ‘cell‐free therapy’ agents as they are responsible for the vast majority of cell‐therapy‐induced beneficent outcomes. Exosomes exert anti‐apoptotic, anti‐fibrotic, cell differentiation, immunomodulatory and pro‐angiogenic effects.^24^, ^25^ It has been reported that the destruction of exosomes by ultrasonication abolishes cardiac progenitor cells (CPCs) angiogenic capacity, demonstrating the importance of exosomes in angiogenic induction.^26^ It has been reported that the beneficial effects of endothelial progenitor cells (EPCs) in endothelial repair may greatly depend on their paracrine impacts in which exosomes play a central role in.^27^ Better performance of CPCs transplantation compared with cardiosphere‐derived cells (CDCs) transplantation in the treatment of myocardial infarction (MI) is mostly due to the greater angiogenesis induction of the CPC‐derived exosomes (two‐fold higher) and higher angiogenic capacity of miRNA cargo of CPC‐derived exosomes.^20^

In this review article, we discussed exosomes and their role in angiogenesis and highlighted recent application of them in ischemic disorders in preclinical models and clinical studies.

## 2 OVERVIEW OF ANGIOGENESIS

Angiogenesis is a complex biologic process attributed to the formation of new vessels in which a variety of cells, mediators and signalling pathways are involved. The central cells involved in the angiogenesis process are endothelial cells (ECs) and pericytes, with their angiogenesis‐promoting dynamics, behaviour and signalling pathways. These cells participate in four fundamental steps of angiogenesis: (a) basement membrane and surrounding extracellular matrix (ECM) degradation, (b) EC proliferation, (c) EC migration and (d) formation of the tubular structures and sprouting.^28^, ^29^

Besides the angiogenesis‐associated cells, several pro‐angiogenic factors such as vascular endothelial growth factor (VEGF), angiopoietins, fibroblast growth factors (FGFs), platelet‐derived growth factor (PDGF) and hypoxia inaudible factor‐1α (HIF‐1α) play crucial roles in neovascularization.^30^, ^31^, ^32^, ^33^, ^34^

Many prominent angiogenesis‐associated factors such as proteins and nucleic acids are incorporated in exosomes, released by special cells and delivered to the recipient cells. Therefore, exosomal content and original cell types impressively affect the angiogenic potential of exosomes.

## 3 EXOSOME: THE CELL OR, CONTENT AND MECHANISM OF ANGIOGENESIS INDUCTION

Exosomes promote angiogenesis by transferring their content into recipient cells. The transferred molecules exert biochemical alterations in the recipient cell, leading to enhanced angiogenic activity.^35^, ^36^ Exosomes are considered ‘mini‐cells’ because they contain multiple bioactive molecules according to their parent cell. Considering the formation process of exosomes, their content is categorized into surface molecules and inner content. Same as a typical cell, the exosomal membrane consists of lipids, carbohydrates and proteins. however, cell‐type‐specific proteins are a wide range of exclusive proteins mediating various therapeutic and pathologic effects of exosomes.^37^

Exosomes' pro‐angiogenic content includes a variety of surface and internal molecules. More prominently, the internal proteins such as VEGF, angiopoietin‐1 (Ang‐1) and heat shock proteins (HSPs), as well as nucleic acids including miRNAs, lncRNAs and circular RNAs (circRNAs) participate in angiogenesis.^38^, ^39^

It is noteworthy that the internalization of exosomes by recipient cells is dependent on multiple factors, including exosome type and recipient cell type. For instance, it has been demonstrated that ECs and cardiac fibroblasts ingest MSC‐derived exosomes with higher amounts compared with cardiomyocytes. Cardiomyocytes uptake EC‐derived exosomes to a greater extent compared with MSC‐extracted exosomes, demonstrating the importance of exosome type. This phenomenon may be partially due to connexins and integrins inserted in the exosomal membrane from different cell types.^40^

After administration of exosomes and subsequent entrance to systemic circulation, exosomes are distributed into tissues.^41^ Following cellular uptake, the endocytic pathway results in breaking down of the exosomal cargo into their metabolites.^42^ Kidneys, liver, spleen and lungs which possess a mononuclear phagocyte system, closely contribute to clearance of exosomes from circulation.^43^ In vivo tracking of exosomes after administration by using sensitive, efficient and biocompatible methods and imaging techniques are highly desired to evaluate the pharmacokinetics of exosomes.^44^ In this regard, pharmacokinetic analysis of gLuc‐lactadherin labelled exosomes by bioluminescent imaging after intravenous injection demonstrated rapid clearance of exosomes with a half‐life about 2 min. Also exosomes were mainly distributed to the liver followed by the lungs.^45^ Consistently after 4 h of IV injection of I^125^ labelled exosomes approximately 1.6%, 7% and 28% of the radioactivity was detected in the spleen, lungs and liver, respectively.^46^

Exosomal content and composition depend on the original cell and the environmental condition. Various cells, including stem cells, mature cells, immune cells and tumour‐associated cells, have been used to isolate exosomes for therapeutic angiogenesis. Stem cell‐derived exosomes could significantly boost angiogenesis and re‐establish blood supply when administered to ischemic areas.^47^ Several stem cell sources have been administered regarding pro‐angiogenic properties, such as MSCs, induced pluripotent stem cells (iPSCs), and adult progenitor cells. It is of crucial importance to note that some factors such as miR‐20 or VEGF receptor‐1 (VEGFR‐1) demonstrate both pro‐angiogenic and anti‐angiogenic properties depending on the type of the recipient cell, dosage and microenvironment.^48^, ^49^, ^50^, ^51^ The pro‐angiogenic content of exsosomes in addition to common cell sources is shown in Table 1.

**TABLE 1:** Angiogenic factors in exosomes, cell source and mechanism of pro‐angiogenic activity.

Angiogenic factor class	Angiogenic factor name	Common cell sources	Pro‐angiogenic mechanism	Reference
Direct angiogenesis stimulant	VEGF	MSC	Boosts ECs proliferation, differentiation, migration, tube formation and sprouting	47
	FGF	MSC	EC proliferation and migration, ECM degradation	47
	EGF	MSC	EC migration, VEGF upregulation	47
	IGF‐1	MSC	EC proliferation, VEGF upregulation	132
	IGFBP‐3	iVPC	Binding and transport of IGF‐1, inducing expression of VEGF, MMP‐2 and MMP‐9	48
	MCP‐2	MSC	VEGF upregulation	47
	PDGF	MSC	ECs proliferation, migration, and sprouting	22
	Ang‐1	MSC	ECs migration, vessel stability	173
	Ang‐2	MSC	ECs sprouting	152
	Flk‐1	MSC	VEGF receptor	173
	ICAM‐1	Nasopharyngeal carcinoma cell	ECs migration	174
	TGF‐β	Breast cancer cell, USC	ECs proliferation	53, 175
	VEGFR‐2	RPE cell, MSC	Main VEGF receptor, promotes ECs proliferation, survival, and migration	30, 47
	BMP‐7	USC	VEGF and VEGF receptors upregulation	53
	EMMPRIN	CPC	VEGF and MMP‐9 upregulation	55
	MMP‐2	Osteoblast	ECM degradation	176
	MMP‐9			
	BDNF	NSC	ECs migration	177
	CTGF	NSC	VEGF upregulation	
	Netrin	NSC	ECs proliferation and migration	
	HMGB1	NCS	VEGF upregulation	
	CA9	RCC cell	Downstream target of HIF‐1α which induces VEGF expression	56
	DMBT1	USC	Binds VEGF and promotes ECs proliferation and migration	67
	PTX3	iVPC	VEGFR‐2 upregulation	48
Heat shock proteins (HSPs)	HSP‐20	Cardiomyocyte	VEFGR‐2 activation	178
	HSP‐70	Endothelial cell	ECs migration and tube formation	179
MicroRNAs	miR‐17‐5p	Nasopharyngeal carcinoma cell	BAMBI suppression	71
	miR‐20b‐5p	iVPC	VEGF overexpression	48
	miR‐21‐3p/5p	MSC, CPC Cardiac telocyte	Stimulates ECs proliferation via activating ERK, Akt, and HIF‐1α	93, 180
	miR‐23a	Gastric cancer cell, MSC	PTEN suppression	181
	miR‐26a	CD34	VEGF, Ang‐1, and MMP‐9 upregulation	50
	miR‐29b‐3p	MSC	PTEN suppression	182
	miR‐31	MSC	HIF‐1α upregulation	80
	miR‐107	Glioblastoma cell	VEGF upregulation	183
	miR‐125a	MSC	DLL4 suppression	77
	miR‐126	MSC, EPC	PTEN suppression, PIK3R2 suppression, PI3K/Akt activation	180, 184
	miR‐130a	MSC	NF‐κB activation	185
	miR‐132	MSC	PI3K/Akt/eNOS signalling activation	137
	miR‐135b	Multiple myeloma cell	HIF‐1α upregulation	79
	miR‐143‐3p	MSC, iVPC	Serpin E1 suppression and VEGF upregulation	48, 180
	miR‐155	M2 macrophage	E2F2 suppression	83
	miR‐181b‐5p	MSC	TRPM7 suppression and HIF‐1α upregulation	78
	miR‐199b‐5p	iPSC‐EC	Jagged1/Notch suppression and VEGFR‐2 upregulation	73
	miR‐210	EPC, Myocyte	VEGF and VEGFR‐2 upregulation	186
	miR‐214	EC	ATM suppression	84
	miR‐221‐3p/5p	M2 macrophage, MSC	PTEN suppression	83
	miR‐384	EPCs	DLL4 suppression	187
	miR‐1246	EPC	ERK1/2 activation	62
Long non‐coding RNAs	lncRNA H19	MSC	VEGF and ICAM‐1 upregulation	40
	lincRNA‐CCAT2	Glioma cell	VEGF and Bcl‐2 upregulation	188
	linc‐POU3F3	Glioma cell	VEGF upregulation	189
CircRNAs	circHIPK3	Cardiomyocytes	VEGF upregulation	125

Mechanism of exosome‐induced angiogenesis could be categorized into three major activities: inducing pro‐angiogenic factors and pathways, preserving the vascular network and regulating the inflammatory response.

### 3.1 Angiogenesis and related molecular pathways

Exosomes could enhance angiogenesis through different mechanisms, including (1) direct transfer of pro‐angiogenic factors into recipient cells, (2) promoting the expression of pro‐angiogenic factors in the recipient cell, and (3) Interfering with the activity of angiogenesis inhibitors:

1. Exosomes transfer pro‐angiogenic factors directly into recipient cells. These factors can influence angiogenesis directly or through regulating central angiogenesis‐related factors. It has been shown that MSC‐extracted exosomes directly transfer Ang‐1 and its receptor, Tie‐2, two central pro‐angiogenic factors, to ECs in order to promote their angiogenesis ability.^52^ Urine‐derived stem cell (USC)‐derived exosomes contain angiogenesis‐related factors, including transforming growth factor‐β1 (TGF‐β1), angiogenin and VEGF, that contribute to exosome‐induced glomerular vascular regeneration and prevention of diabetic nephropathy.^53^, ^54^ Some exosomal contents indirectly promote angiogenesis by enhancing the expression of pro‐angiogenic factors. It has been shown that induced vascular progenitor cell (iVPC)‐extracted exosomes promote cerebral microvascular endothelial cells' (CMVECs') angiogenic capacity through pentraxin 3 (PTX3) and insulin‐like growth factor‐binding protein‐3 (IGFBP3) transfer. PTX3 encourages angiogenesis via upregulating VEGF receptor2 (VEGFR2), while IGFBP3 enhances angiogenesis through IGF‐1R signalling.^48^ CPC and bone marrow mesenchymal stem cell (BM‐MSC)‐derived exosomes improve ECs' angiogenic activity via transferring extracellular matrix metalloproteinase inducer (EMMPRIN) into the ECs which enhance angiogenesis via activation and upregulation of VEGF and matrix metalloproteinase‐9 (MMP‐9). Furthermore, EMMPRIN enhances VEGF signalling via acting as a VEGFR2 co‐receptor.^55^ It has been observed that exosomes derived from renal cell carcinoma (RCC) cells are enriched with carbonic anhydrase 9 (CA9), a downstream target of HIF‐α, and promote human umbilical vein endothelial cell (HUVEC) migration and tube formation through CA9‐mediated MMP‐2 upregulation.^56^
2. Exosomes can alter gene expression of the pro‐angiogenic pathways in the angiogenesis‐related cells. Several angiogenesis‐related pathways are affected by non‐coding RNAs, including extracellular signal‐regulated protein kinase 1/2 (ERK1/2), phosphoinositide 3‐kinase/protein kinase B/endothelial nitric oxide synthase (PI3K/Akt/eNOS), PI3K/Akt/mammalian target of rapamycin (PI3K/Akt/mTOR), signal transducer and activator of transcription 3 (STAT3), mitogen‐activated protein kinase (MAPK), nuclear factor erythroid 2‐related factor 2 (Nrf2), and nuclear factor‐κB (NF‐κB), resulting in upregulation of pro‐angiogenic factors. It has been shown that miR‐126‐enriched‐exosomes derived from BM‐MSCs promote I/R‐injured ECs' tube formation by activating PI3K/Akt/eNOS signalling pathway.^57^ STAT3 is a master transcription factor in the angiogenesis process as it promotes angiogenesis by inducing the expression of VEGF, basic FGF (bFGF), MMP‐2 and MMP‐9.^58^ It has been demonstrated that BM‐MSC‐derived exosomes are enriched in STAT3 and promote HUVEC angiogenic capacity through STAT3 upregulation.^59^

Mitogen‐activated protein kinase is an upstream regulator of ERK that promotes EC proliferation and angiogenic capacity via increasing ERK expression and phosphorylation.^60^ It has been shown that miR‐21‐5p, enriched in USC‐derived exosomes, enhances HUVECs angiogenic activity by boosting MAPK signalling and VEGFR‐1 expression.^61^ It has been reported that umbilical cord blood (UCB) derived EPC‐isolated exosomes improve ECs' proliferation, migration and tube formation via activating ERK1/2 signalling. It has been discussed that promoting the entry of cells into S‐phase of cell cycle via ERK1/2 activation by EPC‐derived exosomes may induce angiogenesis. In addition to ERK1/2, upstream genes including FGF‐2, interleukin 6 (IL‐6) and IL‐8, and some downstream genes, including inhibitor of DNA binding 1 (ID1), cyclooxygenase‐2 (Cox‐2), VEGF, c‐Myc and cyclin D1 were also considerably upregulated.^62^

Tube formation capacity of EPCs is impaired during MI due to C‐X‐C chemokine receptor type 7 (CXCR7) suppression. CXCR7 is a receptor of C‐X‐C motif chemokine 12 (CXCL12); CXCL12, also known as SDF‐1, is a downstream target of Nrf2 and regulates EPCs migration to the ischemic region. Silent mating type information regulation 2 homologue 1 (SIRT1) activates Nrf2; It has been shown that exosomes derived from SIRT1‐overexpressing adipose‐derived MSCs (AD‐MSCs) notably enhance EPCs' migration and tube formation through Nrf2 upregulation and subsequent CXCL12/CXCR7 signalling activation in EPCs.^63^, ^64^

Nuclear factor‐κB improves angiogenesis through induction of VEGF expression. It has been reported that myocyte‐derived exosomes stimulate the NF‐κB pathway by inducing superoxide dismutase 2 (Sod2), probably via miR‐130a transfer. Sod2 is a mitochondrial enzyme that protects the cell from oxidative stress via converting O_2_^−^ into H_2_O_2_.^65^, ^66^ It has been shown that USC‐derived exosomes could promote angiogenesis via deleted in malignant brain tumours 1 (DMBT1)‐mediated activation of PI3K/Akt/mTOR signalling and inducing VEGF expression.^67^

3 Some pro‐angiogenic effects of exosomes are impeding the activity of the angiogenesis‐inhibitor factors and pathways. Non‐coding RNAs target some important angiostatic mediators and pathways, including phosphate and tensin homologue (PTEN), thrombospondin 1 (TSP‐1), delta‐like 4 (DLL4), E2F transcription factor 2 (E2F2), ataxia telangiectasia mutated (ATM) gene, protein tyrosine phosphatase non‐receptor type 9 (PTPN9), transient receptor potential cation channel subfamily M member 7 (TRPM7), receptor tyrosine kinase ligand ephrin‐A3 (EFNA3), Serpin E1 and homeobox proteins growth arrest A5 (HoxA5). Hampering these mediators and pathways finally results in the enhanced expression of angiogenic factors such as VEGF, Ang‐1 and HIF‐1α, as well as the upregulation of cell cycle proteins.

Considering VEGF as a crucial pro‐angiogenic factor, preventing the inhibitors would increase the angiogenesis rate. PTEN is a potent angiostatic gene that suppresses the angiogenesis process by inactivating PI3K/Akt signalling and upregulating the TSP‐1 anti‐angiogenic factor.^68^ It has been reported that miR‐221‐3p of BM‐MSC‐derived exosomes promotes ECs' VEGF levels and angiogenesis through suppressing PTEN expression and subsequent activation of the Akt/eNOS/VEGF pathway.^69^ Through oxygen–glucose deprivation (OGD), miR‐29b‐3p and Akt are downregulated, and PTEN is overexpressed in neurons and brain microvascular endothelial cells (BMECs). It has been shown that BM‐MSC‐derived exosomes transfected with miR‐29b‐3p by lentiviral transfection could promote angiogenesis of OGD BMECs via PTEN suppression, VEGF‐A and VEGFR‐2 upregulation and induced Akt expression in rat model of ischemic stroke.^70^ It has been reported that exosomal miR‐17‐5p extracted from nasopharyngeal carcinoma cells improves ECs' angiogenic activity via suppressing bone morphogenetic protein (BMP) and activin receptor membrane‐bound inhibitor (BAMBI) expression, which abrogates inhibitory effect on Akt/VEGF‐A signalling, resulting in Akt/VEGF‐A upregulation.^71^ PTPN9 is an anti‐angiogenic factor, hampering angiogenesis via inhibiting Akt and ERK phosphorylation and subsequently downregulating VEGFR‐2 expression. It has been shown that miR‐126‐3p and miR‐126‐5p improve HUVECs proliferation, migration and tube formation via PTPN9 repression.^72^ MiR‐210, the primary miRNAs for angiogenesis induction under hypoxic stress, upregulates VEGF and VEGFR‐2 and hampers the EFNA3 activity, leading to augmented angiogenesis induction in ECs. MiR‐130a promotes ECs' tube formation through VEGF and VEGFR‐2 upregulation and hampering anti‐angiogenic factors, including growth arrest homeobox (GAX) and HoxA5.^26^ MiR‐143 enhances angiogenesis via Serpin E1 suppression; Serpin E1, also named plasminogen activator inhibitor‐1 (PAI‐1), hampers angiogenesis through VEGF/VEGFR2 signalling downregulation.^48^ Jagged1/Notch signalling is an angiostatic pathway, inhibiting VEGFR‐2 expression via hairy and enhancer of split 1 (HES1). It has been elucidated that miR‐199b‐5p promotes HUVEC migration, proliferation and tube formation via Jagged1/Notch repression and subsequent VEGFR‐2 upregulation.^73^ RAF1/ERK1/2 signalling enhances angiogenesis via triggering EC proliferation. It has been shown that miR‐126 boosts angiogenesis through silencing sprouty‐related EVH1 domain containing 1 (SPRED1) and phosphoinositide‐3‐kinase regulatory subunit 2 (PIK3R2), which results in RAF1/ERK1/2 upregulation and subsequent VEGF enhanced expression.^74^, ^75^

As with other proangiogenic factors, enhancing angiopoietin and HIF‐1α has a desirable effect on angiogenesis. It has been revealed that miR‐21‐5p, which is abundant in endometrium‐derived MSC (EnMSC)‐extracted exosomes, enhances angiopoietin levels in HUVECs via PTEN suppression and subsequent increased Akt phosphorylation, which leads to VEGF upregulation.^76^ DLL4 is an angiostatic factor that suppresses angiogenesis by prohibiting the formation of endothelial tip cells. It has been revealed that miR‐125a promotes HUVECs proliferation, migration and tube formation by suppressing the DLL4 expression and inducing the expression of Ang‐1 and VEGFR‐2.^38^, ^77^ It has been shown that AD‐MSC‐derived exosomes promote ECs angiogenic capacity under OGD by miR‐181b‐mediated TRPM7 downregulation, leading to increased HIF‐1α expression and decreased expression of tissue inhibitor of metalloproteinase‐3 (TIMP‐3) expression.^78^ MiR‐135b, which is abundant in hypoxic multiple myeloma cell‐derived exosomes, increases angiogenesis via hampering factor‐inhibiting HIF‐1 (FIH‐1). FIH‐1 silencing results in HIF‐1α overexpression, which leads to overproduction of VEGF and Ang‐1.^79^ AD‐MSC‐extracted exosomes promote EC migration and tube formation via miR‐31‐mediated FIH1 inhibition which enhances HIF‐1α transactivation.^80^ HIF‐1α is able to promote EPCs migration to the ischemic areas via CXCL12/CXCR4 enhanced expression and creating a concentration gradient which all lead to improved EPC migration angiogenesis.^81^ Prolyl hydroxylases (PHDs) are enzymes that degrade HIF‐1α in a normoxic condition. It has been reported that miR‐23a promotes angiogenesis via hampering PHD1 and PHD2 activity, resulting in enhanced HIF‐1α levels.^82^

Finally, non‐coding RNAs impress the cell cycle by altering the cell cycle regulators. It has been shown that M2 macrophage‐derived exosomes which enriched in miR‐155‐5p and miR‐221‐5p promote ECs migration, proliferation and tube formation through E2F2 anti‐angiogenic factor downregulation.^83^ It has been shown that EC‐derived exosomes contain high amounts of miR‐214 that suppresses the expression of ATM in recipient endothelial cells. ATM induces senescence and cell cycle arrest, and its downregulation leads to the enhanced angiogenic capacity of endothelial cells.^84^

**FIGURE 1:**
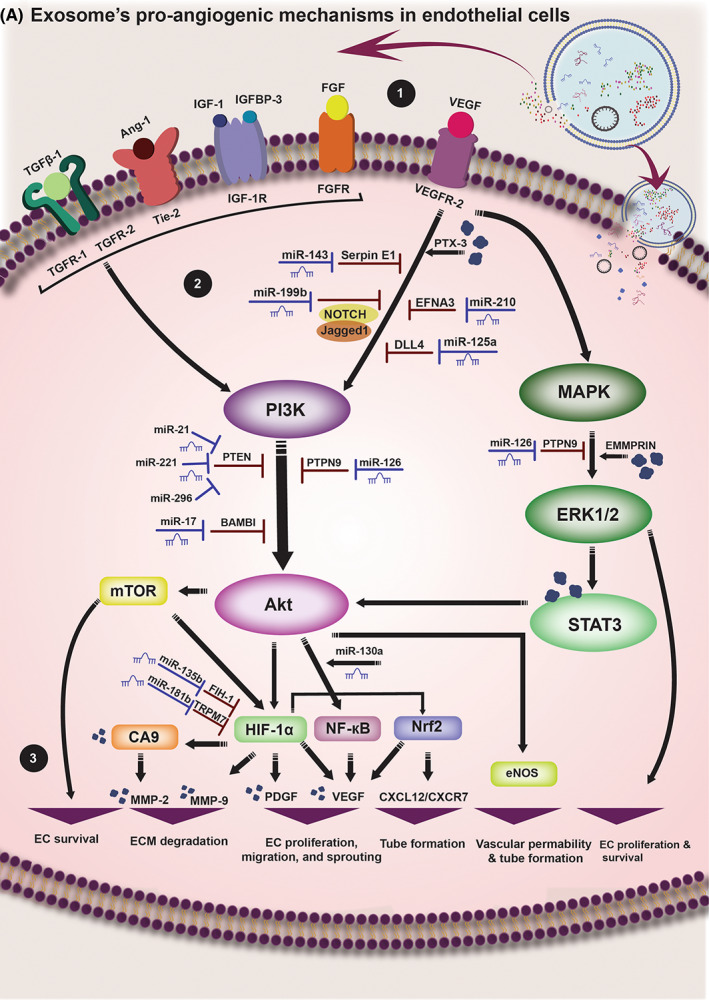
Exosome's angiogenesis induction underlying molecular pathways. 1. Exosomes could release their content outside of ECs. After releasing from exosomes, various angiogenesis stimulants such as VEGF, FGF, IGF‐1, IGFBP‐3, Ang‐1 and TGF‐β1 bind their receptors on the surface of ECs and induce their effects. In addition, exosomes could enter ECs by integration with their membrane or endocytosis and transfer their content into the ECs. 2. Binding of angiogenesis stimulants and their receptors including VEGFR‐2, FGFR, IGF‐1R, Tie‐2 and TGFR‐1,2 initiate a cascade of pathways inside of the ECs. The central factor activated through this process is PI3K. PI3K activates Akt, which subsequently triggers numerous factors including mTOR, NF‐κB, eNOS and HIF‐1α. HIF‐1α then activates CA9 and Nrf2. Exosomal content could boost these signalling via different mechanisms. PTX3 improves VEFFR‐2‐mediated PI3K induction. miR‐130a boosts NF‐κB induction by Akt. Some exosomal content abolish the inhibitory activities of angiostatic mediators; Serpin E1, Jagged1/Notch, EFNA3 and DLL4 prohibit PI3K activation and are, respectively, suppressed by niR‐143, miR‐199b, miR‐210 and miR‐125a. PTEN is a potent inhibitor of PI3K/Akt pathway. Exosomal content such as miR‐21, miR‐221 and miR‐296 silence PTEN and boost PI3K/Akt signalling. PI3K/Akt signalling is also hampered by BAMBI and PTPN9, which are, respectively, silenced by miR‐17 and miR‐126. HIF‐1α is downregulated by FIH‐1 and TRMP7. miR‐135b and miR‐181b upregulate HIF‐1α by inhibiting the activity of FIH‐1 and TRPM7, respectively. In addition, VEGFR‐2 activates MAPK/ERK1/2 signalling. ERK1/2 induces the expression of STAT3 which subsequently activates Akt. Exosomal EMMPRIN boosts ERK1/2 activation; furthermore, miR‐126 suppresses PTPN9, an inhibitor of MAPK/ERK1/2 signalling. 3. The result of angiogenic signalling in ECs is promotion of their angiogenic activity. mTOR enhances EC survival, CA9 induces the expression of MMP‐2 and MMP‐9 that facilitate ECM degradation, MMP‐9 is also induced by HIF‐1α. In addition, HIF‐1α upregulates PDGF. VEGF expression is induced by HIF‐1α, NF‐κB and Nrf2. VEGF and PDGF are the most potent effectors in EC proliferation, migration and sprouting. CXCL12/CXCR7 signalling which is upregulated by Nrf2, facilitates tube formation during angiogenesis. eNOS is responsible for instating vascular permeability and mediating tube formation. ERK1/2 directly enhances EC proliferation and survival.

Taken together, exosomes could be an ideal tool for angiogenesis induction in ischemia‐damaged tissues as they transfer pro‐angiogenic factors into ECs and boost angiogenesis pathways and inhibit angiostatic signalling. In Figure 1, molecular mechanism underlying exosomess' pro‐angiogenic activities are summarized.

### 3.2 Exosomes and preserving vascular network

Ischemia/reperfusion injury, senescence and excessive reactive oxygen species (ROS) disrupt regeneration of vascular networks and need well‐management to increase the stability and functionality of vessels. Studies have suggested that exosomes improve vascular network stability by regulating harmful processes in ischemic tissues.

Ischemia/reperfusion injury results in the cell metabolism shifting to anaerobic metabolism, the adenosine triphosphate (ATP) levels and the intracellular pH reduction, and finally, apoptosis. ECs of newly generated vessels undergo apoptosis through I/R injury, which diminishes the angiogenic capacity needed for ischemic tissue recovery.^85^ Exosomes have shown promising results in maintaining cell viability via activating cell survival pathways, especially PI3K/Akt, and decreasing apoptosis promoters such as p53 to guarantee tissue rehabilitation.^86^

PI3K/Akt pathway is among the most critical survival signalling that prevents apoptosis through various mechanisms, including upregulation of B‐cell lymphoma 2 (Bcl‐2), B‐cell lymphoma‐extra‐large (Bcl‐xL) and survivin anti‐apoptotic factors and downregulating Bcl‐2‐associated X protein (BAX) and Bcl‐2‐associated agonist of cell death (BAD) pro‐apoptotic factors.^87^, ^88^ It has been shown that exosomes extracted from HIF‐1‐modified cardiac ECs possess higher amounts of miR‐126 and miR‐210 that improve CPCs survival under hypoxic stress via increasing ERK and Akt phosphorylation and induce glycolytic switch, leading to improved CPC therapeutic activity post‐MI.^89^ BM‐MSC‐derived exosomes promote I/R‐injured ECs' survival, proliferation and migration, via miR‐126‐mediated activation of the PI3K/Akt/eNOS signalling pathway.^57^

Phosphatase and tensin homologue is a gene that facilitates the apoptosis process through repressing PI3K/Akt signalling and is significantly upregulated during I/R injuries.^90^ It has been shown that BM‐MSC‐derived exosomes inhibit cardiomyocyte apoptosis via miR‐486‐5p‐mediated PTEN silencing.^91^ It has been demonstrated that miR‐29b‐3p suppresses OGD neuron apoptosis through PTEN silencing, resulting in Akt activation and subsequent cleaved caspase‐3 and BAX downregulation and Bcl‐2 upregulation.^70^

It has been shown that miR‐125b‐5p enriched in BM‐MSC‐derived exosomes prevent cardiomyocyte apoptosis during cardiac I/R injury via P53 suppression.^92^ Cardiac telocytes‐derived exosomes hamper ischemic EC apoptosis through miR‐21‐5p‐mediated suppression of cell death‐inducing p53 target 1 (Cdip1), a key downstream target of p53 pathway‐induced apoptosis, leading to caspase‐3 downregulation and improving EC viability which leads in enhanced angiogenesis efficiency and post‐MI recovery.^93^

Ageing is a dominant risk factor for ischemic diseases such as cardiovascular disorders and limb ischemia. ECs undergo the senescence process with ageing, which diminishes their proliferative and angiogenic potential.^94^ During the senescence process, NADPH oxidase‐2 (Nox‐2) expression is upregulated and EC's ROS production is enhanced, leading to increased oxidative stress and making ECs susceptible to impaired angiogenic ability and apoptosis. It is noteworthy that although ROS are essential for VEGF‐induced angiogenesis, excessive ROS amounts have negative impact on the angiogenesis and viability of the ECs. Angiotensin‐converting enzyme 2 (ACE‐2) and eNOS, which are diminished in the senescence process, interfere with oxidative stress. ACE‐2 reduces angiotensin II via converting it to angiotensin I and is known to promote angiogenesis and alleviate oxidative stress. It has been shown that exosomes derived from ACE‐2‐modified EPCs reduce aged ECs' oxidative stress and apoptosis via downregulating Nox‐2 and alleviating ROS generation. Moreover, exosomes improve angiogenic capacity of ECs via eNOS upregulation and subsequently enhanced NO production under I/R injury.^95^, ^96^ It has been shown that co‐culture of EPCs with exosome derived from Nrf2‐overexpressing AD‐MSCs results in enhanced levels of senescence marker protein 30 (SMP30), an anti‐senescence molecule and decreased levels of Nox‐1 and Nox‐4 oxidative stress factors. It seems that activated Nrf2 translocates into the nucleus in order to activate the antioxidant response element (ARE) which induces antioxidant enzyme activity.^97^ Besides, embryonic stem cell (ESC)‐derived exosomes reduce EC oxidative stress via miR‐200a‐mediated suppression of Keap1, a negative regulator of Nrf2.^98^

Excessive ROS production is associated with impaired angiogenesis under hypoxia and OGD. Hence, attenuating oxidative stress could be a positive step in improving angiogenesis and vascular disorders treatment. MiR‐126 protects ECs against apoptosis and oxidative stress by inhibiting ERBB receptor feedback inhibitor 1 (ERRFI1), an inducer of oxidative stress, maintaining cardiomyocyte mitochondrial membrane potential and alleviating intracellular ROS accumulation after I/R injury, resulting in improved cardiomyocyte survival and decreased apoptosis rate.^99^, ^100^ Iron–sulphur cluster scaffold homologue (ISCU) is a direct target of miR‐210. Decreased amounts of ISCU alleviate mitochondrial metabolism and oxygen consumption, leading to diminished mitochondrial ROS production. Decreased metabolic activity is shown to promote cell survival under ischemic stress.^89^ It has been shown that exosomes derived from TIMP2‐modified USCs protect cardiomyocytes from H_2_O_2_‐induced oxidative stress via upregulating oxygen scavenging enzymes including Sod and glutathione (GSH) and downregulation of malondialdehyde (MDA), an oxidative stress marker.^101^

**FIGURE 2:**
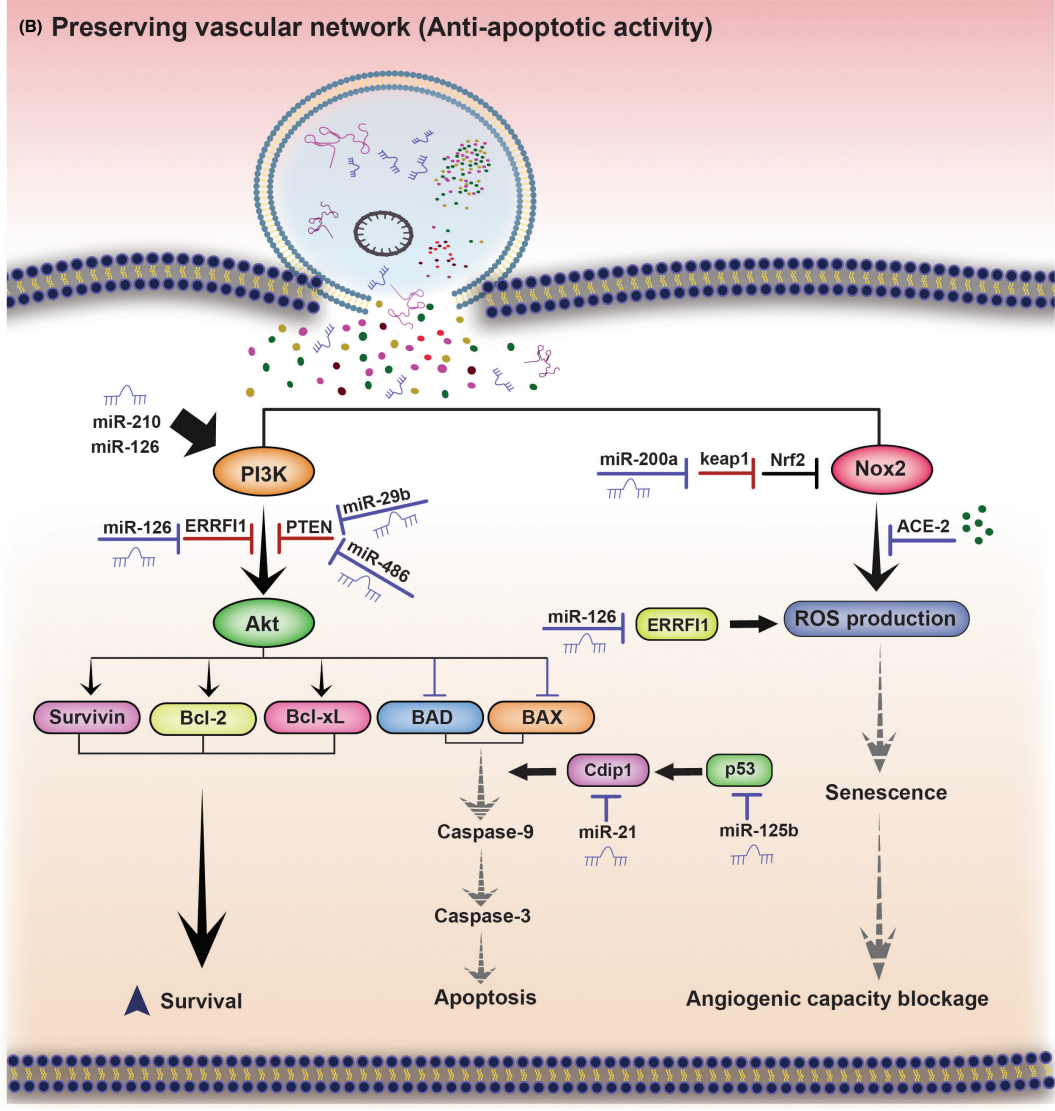
Preserving vascular network (Anti‐apoptotic, anti‐senescence and anti‐oxidative activity) by exosomes. PI3K/Akt is a potent signalling in enhancing cell survival via upregulating anti‐apoptotic factors including survivin, Bcl‐2 and Bcl‐xL and downregulating BAD and BAX apoptotic factors. Exosomal content increase EC survival through various mechanism: miR‐126 and miR‐210 induce PI3K/Akt signalling activation and miR‐29b and miR‐486 abolish the inhibitory effect of PTEN on PI3K/Akt signalling. Similar to PTEN, ERRFI1 hampers the PI3K/Akt signalling, miR‐126 silences ERRFI1, resulting in increased PI3K/Akt activity. p53 and Cdip1 facilitate apoptosis via triggering caspase‐9; miR‐125b and miR‐21 enhance EC's survival via abolishing p53 and Cdip1 effects, respectively. ROS production which is enhanced by Nox2 and ERRFI1, causes oxidative stress, leading to EC senescence and blockage of EC's angiogenic capacity. Exosomal content can modify the senescence process of the EC and hence, improve angiogenesis. MiR‐200a inhibits keap1, resulting in augmented Nrf2 anti‐oxidative activity. ACE‐2 blocks Nox2‐mediated ROS production and miR‐126 hampers ERRFI1 activity, both lead to decreased oxidative stress and cellular senescence

In brief, exosomes are able to diminish ischemic injury and preserve vascular network at the damaged site via inhibiting apoptosis, senescence and oxidative stress in recipient cells. Figure 2 demonstrates exosome's anti‐apoptotic, anti‐senescence and anti‐oxidative mechanism of action.

### 3.3 Exosomes and modulating inflammatory response

Following an ischemic insult, an inflammatory response is occurred in the damaged area due to the apoptosis, oxidative stress and the release of inflammatory cytokines.^102^ The inflammatory response is associated with an increase in various pro‐angiogenic factors, including VEGF, tumour necrosis factor‐α (TNF‐α), TGF‐β, FGF, PDGF, hepatocyte growth factor (HGF), IL‐1α and IL‐6.^103^, ^104^ Macrophages, the most important immune cells through the inflammatory response, possess two main phenotypes: M1 and M2. While M1 is responsible for clearing cell debris and host defence against pathogens, the M2 phenotype primarily participates in tissue regeneration and angiogenesis induction via secretion of pro‐angiogenic factors.^105^ It has been shown that AD‐MSC‐derived exosomes induce M1 to M2 polarization via transport of miR‐21 that targets PTEN and subsequently induces secretion of colony‐stimulating factor‐1 (CSF‐1). CSF‐1 promotes polarization into the M2 phenotype via boosting PI3K/Akt signalling in an autocrine and paracrine manner.^106^ T helper 2 (Th2) cells also enhance angiogenesis via IL‐4 and TGF‐β secretion. BM‐MSC and AD‐MSC‐derived exosomes promote naive CD4^+^ differentiation into the Th2 cells via the transfer of miR‐21 and miR‐29.^103^, ^107^

In summary, exosome therapy could boost tissue regeneration and regulate inflammatory responses in ischemia‐damaged tissues.

**FIGURE 3:**
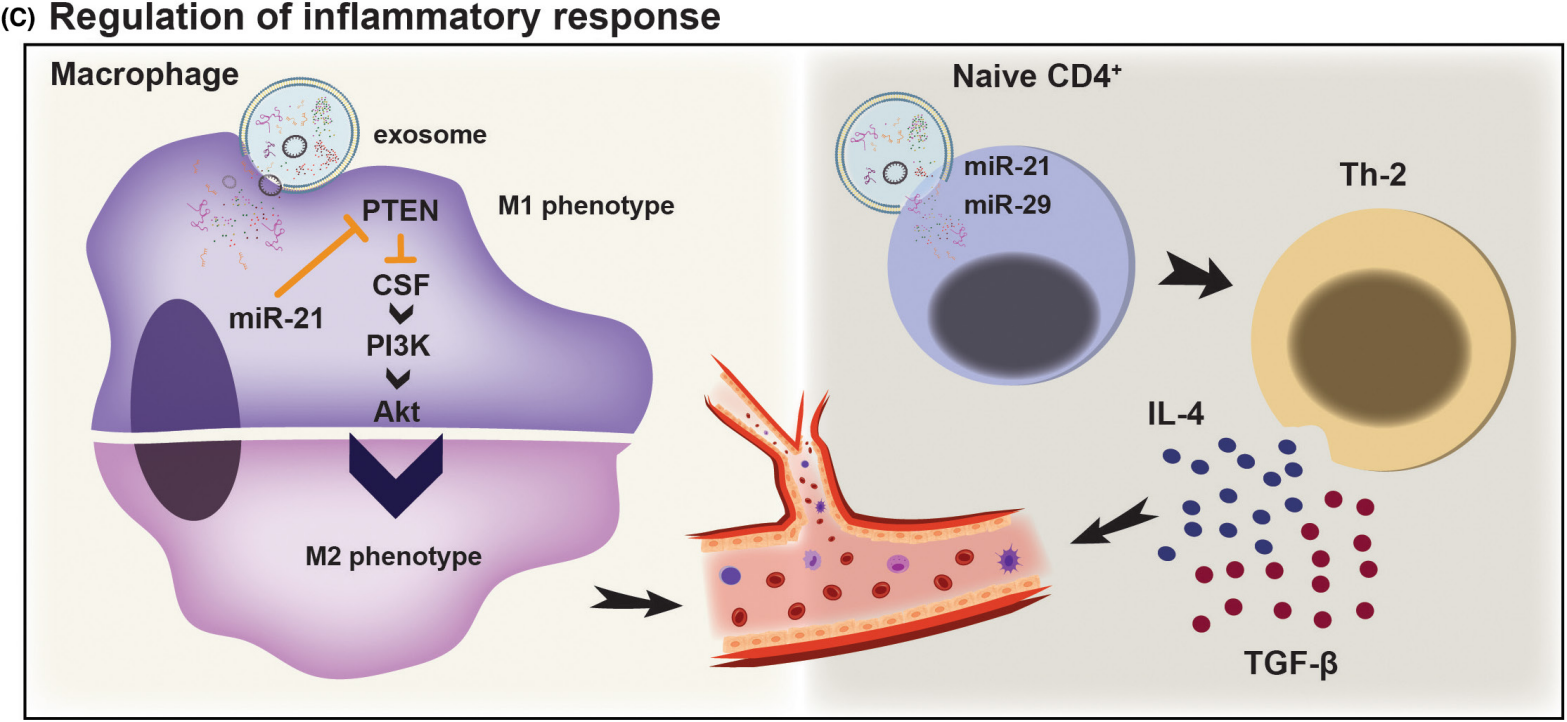
Immunomodulatory mechanisms of exosomes. Regulation of inflammatory response: Exosomes regulate inflammation and exert immunomodulatory impacts in the ischemic damaged area, resulting in increased angiogensis. miR‐21 abolishes the inhibitory effect of PTEN on CSF, leading to activation of PI3K/Akt signalling and M1 to M2 shift of macrophages. In addition, miR‐21 and miR‐29 induce naïve CD4^+^ T cell shift to Th2 cell that secrets IL‐4 and TGF‐β pro‐angiogenic factors. Increase in M2 macrophage and Th2 populations promotes angiogenesis in the ischemic damaged area.

In Figure 3, immunomodulatory mechanisms of exosomes are shown.

## 4 EXOSOMES AND THEIR ROUTE OF ADMINISTRATION

Strategies for delivering exosomes, as therapeutic agents, are an important determinant for a successful therapeutic intervention. The route of administration has a pivotal role in targeting ability, tissue distribution and side effects. In addition, the patient's compliance and the cost of each route are also important to choose the ideal delivery route for each pharmacological agent. Exosomes delivery routes could be categorized into two main categories: systemic and local.^108^

Exosome systemic administration is a cost‐effective and patient‐friendly delivery strategy and is appropriate for systemic disorders such as systemic inflammation and sepsis.^109^ Nevertheless, systemic administration possesses a higher risk of systemic adverse effects and diminishes the exosomes concentration in the target tissue.^110^ Intravenous (IV) injection is the most common systemic administration route used to deliver the exosomes.^111^ While IV administration is convenient and easy, the circulatory short half‐life index of IV‐administered exosomes is a major limitation.^45^ The accumulation of exosomes in the liver and then in the lungs suggests more clearance of exosomes from systemic circulation in IV administration, compared to local routes and other systemic routes.^46^, ^112^ Administration of the exosomes in an intranasal style is considered as a patient‐friendly and non‐invasive strategy. The intranasal administration route might be more effective for retaining the exosomes in the brain tissue compared with IV injection.^113^ The intranasal route diminishes the exosome loss by avoiding the intestinal and hepatic metabolism.^114^

Local administration is described to be useful in several studies, but cannot be used for every organ. Also, the direct injection method seems more efficient in providing a sufficient amount of drug in the target tissue, however, it is more invasive and expensive than a systematic injection of exosomes, as it mostly requires special techniques.^115^ The intramyocardial administration of the exosomes for treatment of cardiac disorders was used in in‐vivo studies for the treatment of cardiologic disorders such as MI.^116^ Intrathecal injection is a local administration route, which is useful for the treatment of spinal disorders. Intrathecal administration of the MSC‐derived exosomes into the mice models of spinal cord injury (SCI) results in improved sensory and locomotor performance by promoting angiogenesis.^117^ Intramuscular (IM) injection is a non‐invasive and frequently used technique for the administration of various drugs. IM injection serves as a proper exosome delivery route for treatment of limb ischemia in animal models.^61^

In conclusion, each delivery strategy pros and cons should be considered and it is up to the investigators and physicians to choose the best strategy for each disease and target organ. Moreover, comprehensive and comparative investigations are required to determine the best exosome delivery route for each disease and condition.

## 5 MODIFICATION STRATEGIES TO IMPROVE EXOSOME FUNCTION

While exosome therapy has shown promising results as a contributory therapeutic tool for the treatment of ischemic disorders, the efficacy needs to be improved to make them an acceptable part of the therapeutic guidelines. Three main strategies that could promote exosome therapeutic potency are preconditioning, gene modification and bioconjugation are highlighted in this section.

### 5.1 Preconditioning

Exosomes' content is extremely affected by the microenvironment in which their origin cells reside.^118^ It has been demonstrated that through myocardial ischemia, cardiomyocytes' exosome production rate, as well as their exosomal pro‐angiogenic content significantly increase.^119^ Preconditioning is a method that simulates various microenvironments such as hypoxic microenvironment, acidic microenvironment, various physical stimuli and presence of diverse biologic and growth stimulations for the cells in order to promote their therapeutic and biogeneration aspects.^120^ Different preconditioning strategies are available such as hypoxic preconditioning, physical preconditioning and preconditioning with drugs and chemicals.^121^ In the setting of exosome generation, it has been shown that various preconditioning strategies could augment a cell's exosome biogenesis as well as enhancing the secreted exosomes' pro‐angiogenic content.^122^

Hypoxic preconditioning is the most utilized strategy in which cells undergo low oxygen tension in their culture milieu or cultured with hypoxia mimetic agents.^123^, ^124^ Under hypoxic stress, HIF‐1α, the most important transcription factor in response to hypoxia, upregulates which enhances the expression of angiogenesis‐related genes such as VEGF and VEGF receptors as well as pro‐angiogenic miRNAs.^26^ It has been demonstrated that hypoxic preconditioning augments the level of pro‐angiogenic factors including VEGF, VEGF‐R2, VEGF‐R3, FGF, monocyte chemotactic protein‐2 (MCP‐2), MCP‐4, Ang‐1, Tie‐2, MMP‐2 and MMP‐9 in MSCs and cardiomyocytes secreted exosomes.^47^, ^116^ It has been shown that hypoxia‐preconditioned cardiomyocytes generate exosomes with higher ability to induce ECs' migration, proliferation and tube formation compared with normoxic cardiomyocytes, mainly due to greater circHIPK3 exosomal content.^125^ Hypoxic preconditioning of CDCs upregulates miR‐126, miR‐130a and miR‐210 content of their exosomes which promote exosomal angiogenic induction in HUVECs in vitro.^26^

Nitric oxide (NO) is a crucial mediator in the angiogenesis process as it promotes EC proliferation, migration and ECM degradation via upregulating bFGF and VEGF.^126^ It has been revealed that MSCs preconditioned with a NO donor, N‐diazeniumdiolates (NONOates), exhibit upregulated miR‐126 and VEGF levels in their secreted exosomes. Moreover, it has been demonstrated that NO‐preconditioned MSCs‐derived exosomes promote HUVECs proliferation, migration and tube formation via VEGFR‐2 and Ang‐1 upregulation.^118^

Drugs could be used as preconditioning inducers and have shown promising results in improving the cell's paracrine potency. It has been revealed that preconditioning of BM‐MSCs with atorvastatin enhanced the levels of lncRNA H19, miR‐675, VEGF and intercellular adhesion molecule‐1 (ICAM‐1) in the secreted exosomes and upregulate PDGF, epidermal growth factor (EGF), bFGF and Ang‐1 in recipient ECs as well as activating the Akt/eNOS pathway.^40^, ^69^ Preconditioning with pioglitazone, an anti‐diabetic medication, is shown to enhance the potency of BM‐MSC‐derived exosomes to hamper PTEN in recipient HUVECs which leads to upregulation of PI3K/Akt/eNOS pathway, resulting in enhanced HUVECs' angiogenic activity.^127^

Physical preconditioning is performed by exposing the cells to physical stimuli such as light and mechanical pressure. It has been shown that human umbilical cord‐derived MSCs (hUC‐MSC) primed with blue light (455‐nm) possess a higher level of miR‐135b‐5p and miR‐499a‐3p pro‐angiogenic miRNAs in their secreted exosomes.^128^ It has been demonstrated that mechanical stress with 15% static stretching could promote the production of exosomes enriched with miR‐1246 pro‐angiogenic factor from fibroblasts.^129^

Taken together, preconditioning of parent cell is a cost‐effective and efficient strategy to improve the quantity and biologic functions of the secreted exosomes. It is crucial to determine the most efficient preconditioning strategy for each cell type and biologic aspect which we intend to promote.

### 5.2 Gene modification, protein and RNA transfection

Genetic modification is a cell manipulation strategy in which the target cell's genome is altered via using various techniques such as viral vectors resulting in DNA sequence alteration and subsequent upregulation or downregulation of specific genes.^130^ As exosome biogenesis and content is proportionate to the parent cell, genetic modification of the parent cell alters its exosome biogenesis and content.

Viruses are appropriate tools for gene modification as they possess a natural instinct to infect the target cell's genome.^131^ It has been shown that induction of glyoxalase‐1 (GLO‐1) overexpression, an enzyme which inhibits extreme accumulation of toxic end products induced by oxidative stress in cells, in MSCs using a lentivirus transfection, improved their produced exosomes VEGF, FGF and IGF‐1 levels. HUVECs cultured with GLO‐1 overexpressing MSCs‐derived exosomes had promoted proliferation, survival, migration and tube formation under high glucose stress in vitro.^132^ In order to enhance exosome targeting ability, MSCs were engineered by lentiviral transfection of ischemic myocardium‐targeting peptide (IMTP) CSTSMLKAC, which resulted in promoted targeting ability and migration capacity of their extracted exosomes to the ischemic myocardium, leading to improved cardioprotective impacts in MI.^133^ It has been shown that induction of HIF‐1α overexpression in MSCs via lentiviral transfection promotes secreted exosomes angiogenic abilities partly via Jagged1 induction.^134^ Lentiviral transfection of CXCR4 gene into the MSCs enhanced CXCR4 level in the secreted exosomes. It has been reported that CXCR‐4 modified‐BM‐MSC‐derived exosomes improve HUVECs angiogenesis via VEGF upregulation.^135^

MicroRNAs could also be transferred via lentiviral transfection. Regarding, it has been indicated that miR‐126‐overexpressing synovium MSCs (SMSCs) which were transfected by miR‐126‐3p lentiviral vector could produce miR‐126‐3p enriched exosomes, which contribute to increased angiogenesis.^136^ It has been shown that miR‐29b‐3p transfected BM‐MSCs secrete exosomes that promote angiogenesis and cell survival via PTEN silencing.^70^ Also, transfection of miR‐132‐3p into the BM‐MSCs via lentiviral vector, enhanced the level of miR‐132‐3p in their secreted exosomes, which activates PI3K/Akt/eNOS signalling in recipient ECs.^137^

It has been shown that induction of Akt gene overexpression in MSCs via an adenovirus transfection system improves their secreted exosomes pro‐angiogenic capacity through enhancing the level of PDGF content.^22^ HIF‐1α is degraded in a normoxic condition and is unable to exert its influence. A research team has designed a mutant HIF‐1α gene which maintains cellular expression in a normoxic environment and transplanted it to the MSCs via an adenoviral transfection system. Mutant HIF‐1α‐modified‐MSCs' secreted exosomes with the capacity to promote recipient BM‐MSCs' proliferation and osteogenic differentiation as well as ECs' angiogenic capacity.^81^

In brief, gene modification and nucleic transfection have shown to minimally influence exosomes' structure and biochemical properties; however, they are associated with high costs, biohazard risks and time consumption which emphasize the necessity of further investigations to shed light on and overcome these obstacles.^63^

### 5.3 Bioconjugation

In bioconjugation, specific biomolecules are included in exosomes.^64^

A strategy to promote exosome targeting ability is conjugating target organ‐specific ligands into the exosome's membrane. It has been shown that there are specific signal molecules on the surface of the exosomes that facilitate exosome uptake by specific tissues.^138^ Conjugation of cyclo (Arg‐Gly‐Asp‐D‐Tyr‐Lys) peptide [c(RGDyK)] onto the exosome surface using bio‐orthogonal copper‐free click chemistry, improves its targeting capability since c(RGDyK) binds to integrin αvβ3 existing in reactive ECs in cerebral vascular network. It has been shown that c(RGDyK)‐conjugated exosomes have a higher migration rate after IV administration to the ischemic brain.^139^ Bio‐orthogonal chemistry method has also been used to conjugate IMTP with hypoxic preconditioned BM‐MSC‐derived exosomes and resulted in profoundly enhanced exosomal migration and retention into infarcted myocardium.^92^ Ischemic regions have a low pH due to high glycolysis rate and low oxygen supply.^140^ It has been demonstrated that conjugation of the intercalated motif (i‐motif), a pH‐sensitive DNA strand enriched with cytosine, significantly promotes exosomes delivery to acidic areas, which could promote exosome targeted‐delivery to ischemia‐bearing sites.^141^ In another study, hyaluronic acid grafted with 3‐diethylamino propylamine (HDEA) was loaded into exosomes via sonication, a physical method to load cargos into exosomes via creating pores in the exosomal membrane by ultrasonic waves; it has been shown that membrane of HDEA‐loaded exosomes significantly desbalized in pH = 6.5, which resulted in releasing their content in an acidic environment.^142^

Nanoscale cargos such as miRNAs could be loaded into the exosomes via incubation. It is possible to load miR‐210 into the MSC‐derived exosomes via cholesterol modification which creates lipophilic miRNAs that could efficiently emerge with exosome membrane; incubation of exosomes with lipophilic miR‐210 enhances exosomal miR‐210 level, which results in improved pro‐angiogenic capacity.^143^ Electroporation is another strategy to load biologic substances into the exosomes. Electroporation utilizes electrical flow to notch exosomal membrane in order to create micro‐pores and enhance exosomes' permeability, thus facilitating molecular penetration into exosomes. It has been reported that electroporation of miR‐132 into the MSCs‐derived exosomes significantly enhances their angiogenesis induction in HUVECs.^23^ Electroporation possesses a higher efficacy in loading cargos into the exosomes compared with incubation, but it is also associated with a higher risk for manipulating and disrupting the exosome' structure as well as a more complex procedure.^144^

**FIGURE 4:**
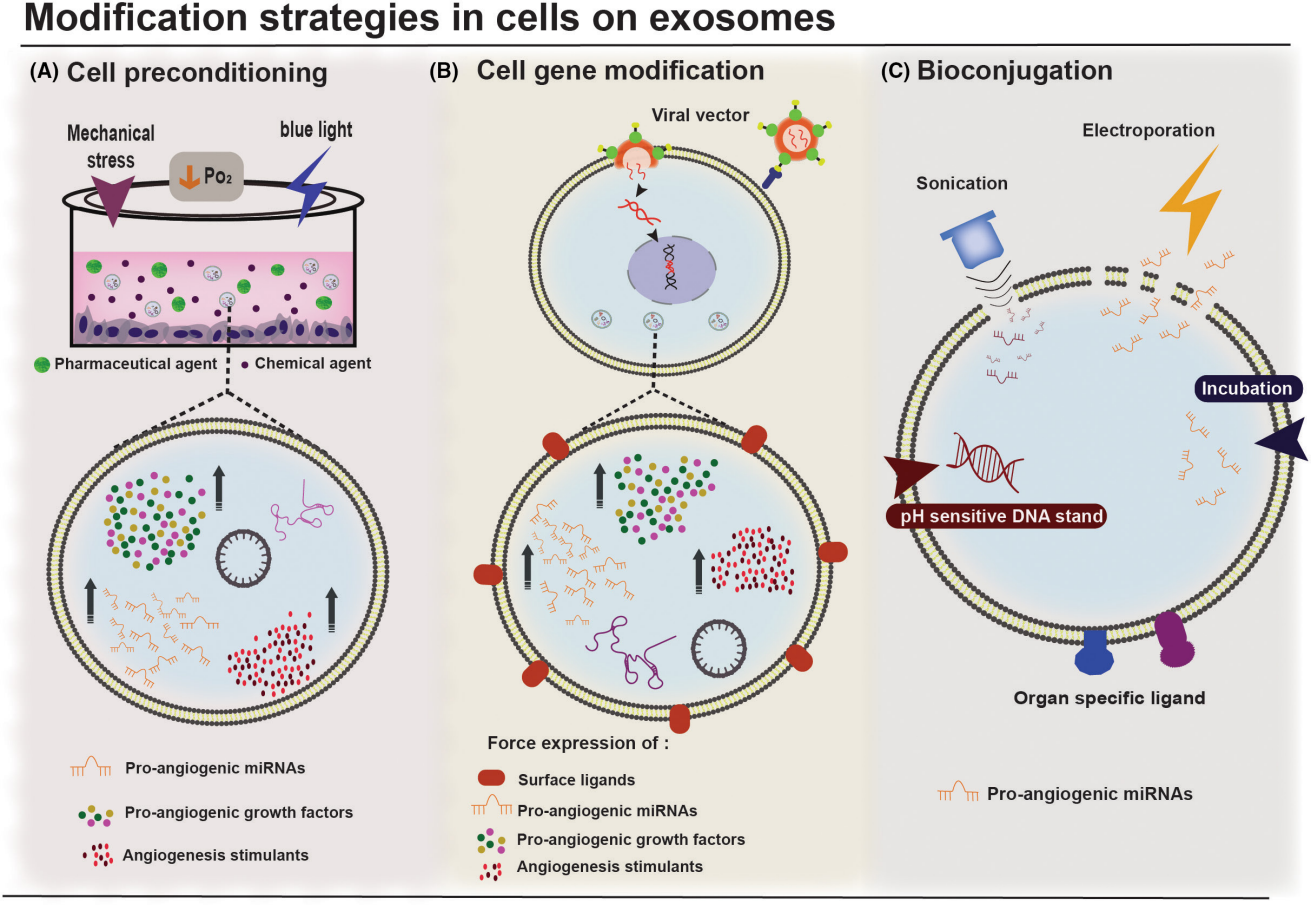
Strategies to improve exosomes therapeutic function. Modification strategies to enhance exosomes therapeutic potential for the treatment of ischemic disorders can be categorized in three groups: (A) Cell preconditioning: Treatment of the parental cell with various preconditioning inducers such as blue light, hypoxia (↓P_O2_), mechanical stress, pharmaceutical agents and chemical agents modifies intracellular pathways involved in angiogenesis, resulting in enhanced exosome biogenesis and exosomal content of pro‐angiogenic miRNAs, growth factors and stimulants. (B) Cell gene modification: A specific angiogenesis‐related gene is inserted into the parental cell, commonly via a viral vector, and enhances the expression of organ‐specific surface ligands, pro‐angiogenic miRNAs, growth factors and stimulants. (C) Bioconjugation: Organ‐specific surface ligands, pH‐sensitive DNA strands and pro‐angiogenic miRNAs and stimulants are conjugated with exosomes through different methods including incubation, sonication and electroporation.

In Figure 4 different modification strategies to improve exosomes therapeutic capacity and their effects are shown.

## 6 CLINICAL APPLICATION OF EXOSOMES, A PERSPECTIVE OVERVIEW

Although a huge amount of evidence has elucidated various therapeutic potentials of exosomes in ischemic disorders, there are several challenges in the safety and efficacy of exosome therapy which need to be overcome.^145^ Appropriate preclinical models are important to evaluate the pharmacodynamics, pharmacokinetics and toxicology of exosome as a novel drug. Indeed, preclinical studies could elucidate efficacy, potency and safety of exosomes, thereby increasing the chances of successful translation into clinical setting.^146^ However, most diseases are often complicated by multifactorial aetiologies which affect clinical management and are not predictable in animal model studies with are conducted in a uniform genotype. This issue generally results in a trade‐off between convenience and physiological applicability.^147^ In this way, safety, efficacy, potency and efficient dosage identified in animal studies are generally not translated to clinical trials.^148^ To date, a magnificent number of in vivo studies have been conducted to evaluate the safety and efficacy of exosome therapy. Neurological, cardiovascular and immune‐related diseases represent the three most investigated areas for exosome therapy. However, a significant number of studies used musculoskeletal, liver, kidney and pulmonary diseases animal models.^149^ A considerable number of experimental studies on exosomes demonstrated that exosomes have a promising therapeutic potential for ischemic diseases. Application of exosomes in ischemic diseases may contribute with multiple advantages, which mostly refer to containing pro‐angiogenic factors and/or regulating the survival signalling pathways.^150^ Besides, protecting against I/R injury and oxidative stress, as well as regulation of immune response, apoptosis and necrosis contribute to the beneficial effects of exosomes in the treatment of ischemic disorders.^47^, ^80^, ^151^

Preclinical investigations in terms of ischemic diseases mostly explored cutaneous wound healing,^67^ skin burn injury,^152^ flap transplantation in the treatment of refractory wounds,^153^ fat grafting,^151^ MI,^99^ limb ischemia,^132^ ischemic stroke,^70^ hepatic I/R injury,^154^ and retinal ischemia.^155^ In addition, it has been found that exosomes may play an important role in the treatment of several diseases which could develop following ischemia and ischemia‐related conditions such as cardiac fibrosis,^24^ bone defects,^156^ osteoporosis,^38^ osteonecrosis of the femoral head (ONFH),^50^ SCI,^117^ renal fibrosis^157^ and acute kidney injury (AKI).^158^ In vivo studies regarding ischemic diseases were described in detail in Table 2.

**TABLE 2:** In vivo studies of exosome applications for the treatment of ischemic disorders classified by disorders type, cell origin, mechanism of therapeutic action and route of delivery.

Category	Type of disease	Cell origin	Mechanism of action (target signalling pathway)	Rout of delivery	Reference	Effect
Skin	Diabetic wound	UCB‐derived EPC	ERK1/2 activation	Subcutaneous	62	Improved wound closure Reduced the ulcerated area Increased formation of the granulation tissue Enhanced healing speed
		Nrf2‐Overexpressing AD‐MSC	Nrf2 overexpression and VEGF, Nox‐1 and Nox‐4 upregulation	Local injection	97	
		UCB‐derived EPC	FGF‐1, VEGF‐A, VEGFR‐2, Ang‐1, E‐selectin, CXCL‐16, eNOS and IL‐8 overexpression	Subcutaneous	190	
		Melatonin‐primed BM‐MSC	PTEN suppression /Akt activation	Subcutaneous	191	
		USC	DMBT1 protein transfection, subsequently VEGF‐A overexpression and PI3K/Akt activation	Subcutaneous	67	
		Deferoxamine‐preconditioned BM‐MSC	miR‐126‐mediated PTEN/PI3K/Akt activation	Subcutaneous	192	
		hUC‐MSC	VEGF and TGFβ‐1 upregulation	Topical application	54	
		hUC‐MSC	ERK1/2 activation and VEGF upregulation	Topical application	193	
		miR‐126‐3p‐overexpressed SMSC	miR‐126‐3p‐mediated PI3K/Akt and MAPK/ERK activation	Topical application	136	
	Aged pressure ulcer	ESC	miR200a‐mediated Keap1 downregulation and Nrf2 activation	Topical application	194	Accelerated pressure ulcer healing
	cutaneous wound	hiPSC‐MSC	OCN, Sox9, and LPL genes overexpression and collagen synthesis promotion	Subcutaneous	195	Accelerated re‐epithelialization Enhanced wound healing
		AD‐MSC	lncRNA MALAT1‐mediated increased cell migration	Topical application	196	
	ischemic auricular wound	Platelet exosome product	TGF‐β‐related downstream pathways activation, and promotion of collagen organization	Topical application	175	Enhance overall skin tissue organization Facilitate ischemic wound healing
		Placental‐derived MSCs	HGF, IGFBP‐2, IGFBP‐3, and IGFBP‐6 upregulation	Subcutaneous	52	
	second‐degree burn injury	hUC‐MSC	Wnt4/ β‐catenin activation	Subcutaneous	150	Improved cutaneous wound healing Improved cutaneous regeneration
		Blue light preconditioned‐MSC	miR‐135b‐5p and miR‐499a‐3p‐mediated MEF2C gene suppression	Subcutaneous	128	
	Skin flap ischemia	H_2_O_2_‐preconditioned AD‐MSC	Promotion of EC proliferation, and alleviation of inflammation and apoptosis	Subcutaneous	153	Enhanced survival of the skin flap Reduced the skin flap in I/R injury Enhance epithelialization
	fat graft	AD‐MSC	Promotion of early inflammation, angiogenesis, adipogenesis and collagen synthetize	Subcutaneous	197	Improved fat retention Decreased fibrosis
Cardiology	Acute myocardial infarction	Cardiac‐MSC	Enhancement of capillary tube formation	Intramyocardial	198	Protecting myocardium against I/R injury Improved cardiac function and EF Reduced cardiac fibrosis Decreased infarction size Decreased cardiac remodelling Improve LV contractility Facilitating cardiac regeneration
		hUCMSC	Bcl‐2 upregulation	IV	199	
		Hypoxia‐preconditioned BM‐MSC	miR‐125b‐5p‐mediated p53 and BAK1 genes suppression	IV	200	
		BM‐MSC	miR‐29c‐mediated PTEN/Akt/mTOR activation	Intramyocardial	201	
		iPSC	Nanog‐regulated miR‐21 and HIF‐1α‐regulated miR‐210‐mediated caspase inactivation, apoptosis inhibition and cardio‐protection	Intramyocardial	202	
		Akt‐modified hUCMSC	PDGF‐D/PDGFR activation	IV	22	
		Atorvastatin‐preconditioned MSC	LncRNA H19 and miR‐675‐mediated VEGF and ICAM‐1 upregulation	Intramyocardial	40	
		miR‐132‐electroporated MSC	miR‐132‐mediated RASA1 gene downregulation	Intramyocardial	23	
		HIF‐1α overexpressing MSC	HIF‐1α‐mediated PDGF and VGEF upregulation	IV	34	
		CXCR4‐modified MSC	CXCR4‐mediated PI3K/Akt activation and VEGF upregulation	Patch graft in infracted zone	135	
		Hypoxic Preconditioned cardiomyocyte	CircHIPK3‐mediated VEGF‐A over expression	Intramyocardial	125	
		AD‐MSC	miR‐31‐mediated FIH1/HIF‐1α transcriptional activation	IV	80	
		
		Dendritic cells co‐cultured with hypoxic cardiomyocytes	miR‐494‐3p‐mediated VEGF overexpression	Intramyocardial	203	
		TIMP2‐modified UC‐MSC	Akt/ Sfrp2 upregulation, and MMP‐2 and MMP‐9 downregulation	Intramyocardial	101	
		Plasma of remote ischemic conditioned models	HSP70‐mediated eNOS, iNOS, HIF‐1α, Ang‐1 and VEGF overexpression	IV	39	
		Cardiac telocyte	miRNA‐21‐5p‐mediated Cdip1/Caspase‐3 downregulation	Intramyocardial	93	
		EnMSC	miR‐21‐mediated PTEN suppression	Intramyocardial	76	
		SIRT1‐ overexpressing AD‐MSC	SIRT1‐mediated Nrf2/CXCL12/CXCR7 upregulation	Intramyocardial	204	
		Cardiomyocytes	miR‐222 and miR‐143‐mediated MMP‐2 and MMP‐9 increase	Intramyocardial	116	
		GATA‐4 overexpressed MSC	miR‐19a‐mediated PTEN suppression and Akt/ERK activation	Intramyocardial	205	
	Cardiac fibrosis	Human amniotic fluid‐derived MSCs	HIF‐1α and VEGF upregulation	IV	24	Decreased collagen I and ECM deposition in the heart
	Diabetic cardiomyopathy	HSP20‐transgenic cardiomyocytes	Hsp20, p‐Akt, Sod1, and survivin upregulation	IV	178	Improving cardiac function Decreasing hypertrophy and fibrosis
Bone	Bone fracture	Hypoxia preconditioned hUC‐MSC	miR‐126‐mediated SPRED1 suppression and RAS/ERK activation	Local injection	206	Enhanced fracture healing
	ONFH	hiPS‐MSC	PI3K/Akt activation	IV	207	Prevent bone loss and ONFH development Accelerated bone regeneration Protect femoral head from necrosis Promoting osteogenesis
		hypoxia‐preconditioned BM‐MSC	VEGF overexpression	IV	208	
		CD34^+^ stem cell	miR‐26a‐mediated promotion of ECs migration and tube‐formation	IV	50	
	Calvaria critical‐sized defect	DMOG‐preconditioned BM‐MSC	PTEN suppression / Akt/mTOR activation	Local implantation	209	Improve bone regeneration and healing Induce mineral deposition and osteogenesis
		HIF‐1α‐modified BM‐MSC	HIF‐1α‐mediated RUNX‐2, ALP and collagen I overexpression	Local implantation	81	
		hiPSC‐MSC	RUNX‐2, collagen I and ALP overexpression	Local implantation	210	
	Avascular necrosis of femoral head	HIF‐1α‐ modified BM‐MSC	HIF‐1α‐mediated OCN and ALP overexpression, and promotion of ECs proliferation and tube formation	Local injection	211	Accelerated bone regeneration improve reconstruction in femoral heads necrosis area
	Osteoporosis	miR‐29a‐loaded BM‐MSC	miR‐29a‐mediated VASH1 downregulation	IV	38	Improve osteogenesis Prevent osteoporosis progression Increased trabecular bone mass Improve bone blood flow
		BM‐MSC	LncRNA H19‐mediated miR‐106a inhibition and Ang‐1/Tie2‐NO activation	IV	212	
Limb ischemia	Hind limb ischemia	Cardiac‐MSC	miR‐7116‐5p‐mediated protein polyubiquitination inhibition	IM	213	Improved limb perfusion Improve motor function Promote ischemic tissue repair Reduced incidence of ischemia‐induced necrosis Fibrosis tissue reduction Protect limbs from ischemic injury Diminishing cellular senescence
		Human CD34^+^ cells	miR‐126‐3p‐mediated SPRED1 suppression	IM	214	
		Glioblastoma	miR‐221‐mediated FGF‐2 and Ang‐2 upregulation	Local delivery	183	
		AD‐MSC	miR‐21‐mediated CSF‐1R and PI3K/Akt activation	IM	106	
		AD‐MSC	miR‐31‐mediated FIH1/HIF‐1α transcriptional activation	IM	80	
		Myocardial ischemia patients' serum	miR‐939‐mediated iNOS‐NO activation	IM	119	
		NO‐preconditioned human placenta‐derived MSCs (hP‐MSCs)	miR‐126‐mediated NO stimulation and PI3K/Akt activation	IM	118	
		hP‐MSC	VEGF‐A, VEGFR‐2, Ang‐1, Ang‐2, and eNOS overexpression	IM	215	
		iVPC	IGFBP‐3 and PTX3 upregulation	IM	48	
		hiPSC	VEGF, TGF‐β1 overexpression	IM	216	
	Hind limb ischemia of diabetic foot	MSC	miR‐21‐5p‐mediated Akt and MAPK activation	IM	61	Enhanced perfusion Increased integrity of muscle structure
		GLO‐1 overexpressing AD‐MSCs	eNOS/Akt/ERK/MAPK activation	IM	132	
CNS disorders	Spinal cord injury	NSC	VEGF‐A upregulation	IV	177	Reduced the lesion area Improved locomotor and sensory function Enhanced neurogenesis
		MSC	BAX suppression/Bcl‐2 upregulation, TNF‐α and IL‐1β downregulation, IL‐10 upregulation	IV	16	
		miR‐126‐modified MSC	miR‐126‐mediated SPRED1/PIK3R2 suppression	IV	217	
	Traumatic brain injury	BM‐MSC	Increased neurogenesis, angiogenesis and reduced inflammation	IV	218	Improved cognitive and sensorimotor functional recovery Increased brain neurogenesis Diminished neurological deficits
	Ischemic stroke	miR‐210‐loaded MSC	miR‐210‐mediated VEGF overexpression	IV	143	Reduced infarcted area Neuroprotection Promoted neurological and neurobehavioral recovery function Reduced microglia and neuron pyroptosis Attenuated BBB dysfunction Amelioration of the hypoxic–ischemic brain injury
		AD‐MSC	miR‐126‐mediated neurogenesis	IV	219	
		MSC	miR‐132‐3p‐mediated RASA1/RAS/PI3K/Akt/eNOS activation	IV	137	
		BM‐MSC	miR‐26b‐3p‐mediated PTEN suppression /Akt activation	Stereotactic injection	220	
		EPC	miR‐126‐mediated VEGFR‐2 upregulation	IV	221	
		CXCR4‐overexpressing BM‐MSC	Wnt3a/β‐catenin inactivation	Stereotactic injection	222	
Renal disorders	AKI	Endothelial colony forming cell (ECFC)	Attenuation of hypoxia‐induced ECs apoptosis	Direct injection into renal arteries	223	Decreased tubular necrosis Promoted recovery from I/R renal injury Decreased creatinine levels Improved kidney function
		ECFC	miR‐486‐5p‐mediated PTEN suppression/ Akt activation	IV	224	
		Melatonin‐Preconditioned MSC	Increased HO‐1 gene expression, and Sod, CAT, GPX activities Decreased caspase‐3 activity and PARP1 BAX genes expression Bcl‐2 gene upregulation IL‐10 upregulation bFGF, HGF, SOX9 and VEGF upregulation	Direct injection into renal arteries	225	
	Tubulointerstitial fibrosis	GDNF‐modified AD‐MSC	SIRT1/eNOS activation	IV	157	Diminished renal fibrosis Improved kidney function Ameliorate peritubular capillary loss
	Diabetic nephropathy	USC	BMP‐7‐mediated VEGF and TGF‐β overexpression	IV	53	Improved renal regeneration Prevent diabetic kidney injury
Hepatic disorders	Hepatic I/R injury	hiPSC‐MSCs	Decreased TNF‐α, IL‐6 and HMGB1 expression Caspase‐3 and BAX downregulation Increased GSH, GSH‐Px and Sod expression	IV	154	Alleviated hepatic I/R injury Reduced levels of plasma AST and ALT Diminished hepatocyte necrosis, sinusoidal congestion and cell swelling
Retinal disorders	Retinal Ischemia	BM‐MSC	CREB and HSP activation	Intravitreal	155	Preservation of retinal vascular flow Protection against retinal I/R injury

Accordingly, the safety and efficacy of exosomes administration in pathological status are approved by preclinical and animal model studies, it is critical to evaluate safety and efficacy of exosomes usage under three phases of clinical trials, before their approval for clinical utilization.^159^ In this regard, there are several valid acceptable standards for controlling quality of novel products including European Medicines Agency (EMA), Food and Drug Administration (FDA) and Health Canada, that provide certain guidelines to approve a novel drug for administration.^160^ To date, the majority of clinical trials are related to exosome utilization as early diagnostic tools or predictors of treatment outcomes and clinical trials for therapeutic usage of exosomes are limited. In the terms of main issues in clinical use of exosomes, it could be referred to providing the optimal cell culture conditions, protocols for exosome production, isolation and storage, optimal dose for humans, schedule of exosomes administration, choosing the proper route of administration, and developing a protocol for modification strategies in order to promote exosomes therapeutic potential.^161^ Most of the published clinical studies showed beneficial effects of exosome administration without serious adverse effects in cancers including melanoma,^162^ non‐small cell lung cancer^163^ and colon cancer.^164^ Table 3 summarized the published and ongoing studies regarding ischemic diseases.

**TABLE 3:** Completed and ongoing clinical trials on exosome‐induced angiogenesis for ischemic disorders

Type of disease	Source	Dose	Administration route	Results/status	Reference
Acute ischemic stroke	Placenta‐derived MSC	200 μg	Stereotaxic injection	Safe Without serious adverse events Promoted motor function	226
Chronic kidney diseases	hUC‐MSC	100 μg/kg/dose	Two doses: intraarterial and intravenous	Safe, well‐tolerated Improved kidney function decreased inflammation	227
Skin wound	Plasma	Not reported	Local application	Unknown status	NCT02565264
Diabetic cutaneous ulcer	MSC	Personalized nutritional Intervention	Oral	No result	NCT05243368

## 7 CONCLUSION AND FUTURE PERSPECTIVE

Although exosomes have been extensively investigated as therapeutic modalities for ischemic diseases, there are still many challenges which need to be addressed, mainly in terms of optimization and improvement of isolation protocols and effective dose escalation.

One of the most important challenges that exosome‐based therapies have faced is the rapid clearance and short half‐life of exosomes in vivo. To produce practical scale of exosomes, scale‐up in vitro cell culture systems should be established. This is a considerable challenge for health experts. A technique to enhance exosome production is application of three‐dimensional (3D) culture system which supports better cell‐to‐cell communication and promotes exosome biogenesis.^165^ It has been demonstrated that hUC‐MSCs cultured in a 3D condition possess a 19.4 folds higher exosome production compared to the hUC‐MSC cultured in 2D condition.^166^ Moreover, exosomes could be used in combination with conventional therapies. For instance, integration of MSC‐derived exosomes into scaffolds and hydrogels could significantly improve the wound healing process via promoting angiogenesis and inflammation regulation.^167^ Exosome can also serve as an ideal vehicle for drug delivery. Various drugs as chemotherapeutics and angiogenesis‐stimulators could be loaded on exosomes and delivered different biomedical components to target tissues with great efficacy.^168^ As naked exosomes undergo extensive phagocytosis and clearance shortly after transplantation, it has been shown that embedding exosomes on biomaterials such as stents, cardiac patches and cell sheets could profoundly enhance their sustainability and therapeutic efficacy.^169^

Tumour‐associated exosomes contain considerable angiogenic molecules since angiogenesis is a crucial necessity for tumour development, expansion and far metastasis. It is known that cancerous cell‐derived exosomes participate in tumour angiogenesis; thus, neoplastic cells could be suitable sources for angiogenic‐exosome isolation.^68^, ^170^ Tumour cell characteristics exert an essential impact on the properties of secreted exosomes. For instance, chemoresistant ovarian cancer cell‐derived exosomes possess more powerful angiogenic impacts than those derived from normal ovarian cancer cells.^171^ Nevertheless, utilization of cancerous cells for exosome extraction may increase the risk of carcinogenesis in the target site.^172^ In conclusion, exosomes could be an ideal therapeutic tools for the treatment of ischemic disorders due to their significant pro‐angiogenic capacity and unique biological properties. However, for a prosperous clinical translation, it is crucial to optimize their therapeutic activity, define certain protocols for extraction, modification and administration, as well as conducting more investigations on their molecular mechanism of action.

## AUTHOR CONTRIBUTIONS

**Kasra Moeinabadi‐Bidgoli:** Conceptualization (equal); data curation (equal); investigation (equal); project administration (equal); software (equal); writing – original draft (equal); writing – review and editing (equal). **Maliheh Rezaee:** Data curation (equal); writing – original draft (equal); writing – review and editing (equal). **Nikoo Hossein‐Khannazer:** Conceptualization (equal); project administration (equal); writing – review and editing (equal). **Amirhesam Babajani:** Conceptualization (equal); writing – original draft (equal). **Hamid Asadzadeh Aghdaie:** Conceptualization (equal); project administration (equal). **Mandana Kazem Arki:** Visualization (equal). **Siamak Afaghi:** Data curation (equal); writing – review and editing (equal). **Hassan Niknejad:** Conceptualization (equal); supervision (equal); writing – review and editing (equal). **Massoud Vosough:** Conceptualization (equal); investigation (equal); project administration (equal); supervision (equal); validation (equal); visualization (equal); writing – review and editing (equal).

## FUNDING INFORMATION

None.

## CONFLICT OF INTEREST STATEMENT

The authors confirm that there are no conflicts of interest.

## CONSENT FOR PUBLICATION

Not applicable.

## Data Availability

Data sharing is not applicable to this article as no new data were created or analyzed in this study.
